# Fighter, Corpsman, Partisan an Attempt to Typify Former Soldiers Based on their Coping and Defense Mechanisms

**DOI:** 10.1007/s12124-019-09507-1

**Published:** 2019-12-02

**Authors:** Loni Brants, Katrin Schuy, Simone Dors, Marie Horzetzky, Heinrich Rau, Gerd Willmund, Andreas Ströhle, Stefan Siegel

**Affiliations:** 1 Department of Psychiatry and Psychotherapy, Campus Charité Mitte, Charité – Universitätsmedizin Berlin, corporate member of the Freie Universität Berlin, Humboldt-Universität zu Berlin, and Berlin Institute of Health, Charitéplatz 1, 10117 Berlin, Germany; 2 Psychotraumazentrum, Military Hospital, 10115 Berlin, Germany

**Keywords:** Veterans, Former soldiers, Trauma, Coping, PTSD, Defense, GAF, Qualitative survey

## Abstract

This work strives to develop a typological classification of the use of conscious and unconscious defense and coping mechanisms based on methodically and structurally collected data from a qualitative survey of 43 former soldiers in Germany. Seven coping and defense types were identified: *the Fighter, the Comrade, the Corpsman, the Strategist, the Partisan, the Self-Protector* and *the Infantryman*. The types identified differed with regard to the accumulation, combination, and use of their conscious and unconscious defense and coping mechanisms in the superordinate areas of *behaviour, relationships, emotions, reflexivity* and *time focus*. The typological classification could offer psychotherapeutic interventions tailored to individuals and their defense and coping mechanisms, which could lead to improved therapy use and compliance.

## Introduction

### The Situation of a Former German Soldier in an International Comparison

In all, 85.5% of the German Armed Forces’ (GAF) foreign missions are associated with the experience of stressful events and an increased prevalence of mental illness with a generally low level of therapy use (Wittchen et al. 2012). In addition to traumatizing events during their missions abroad and experiences of discrimination at home due to their careers, (former) soldiers in general have to manage high amounts of stress and adjustment. Different demands (assimilating into the military system, war experiences abroad, return and reintegration into professional and family life afterwards, leaving the military, reintegration into civilian life and perhaps dealing with symptoms of a trauma-related disorder) require an extremely high degree of adaptability (Schuy et al. 2018¸ Brants et al. 2018). In contrast to the broad state of knowledge about the mental health of, for example, US soldiers, little is known about the handling of stressful events and mental illness among *active* GAF soldiers (Kowalski et al. 2012), and even less is known about the coping of *former* GAF soldiers who have left the military system (Siegel et al. 2017). For various reasons, it is not possible to easily apply international findings to the situation in Germany, not least because of diverging national socio-cultural factors and different benefits laws. The attitudes of the German public to military forces in general, and the GAF in particular, are still influenced by Nazi history and the war crimes of the German Wehrmacht. Therefore, active and former German soldiers, especially mentally ill soldiers, are exposed to high levels of stigmatization, which they have to cope with in their everyday life and which makes it even more difficult to seek professional help (Schuy et al. 2018). However, even in countries such as the USA, where traumatization of soldiers and their (subsequent) coping mechanisms have been the focus of scientific and societal attention for some time now, to our knowledge, there are hardly any studies or publications on the defense *and* coping mechanisms of former soldiers.

### Coping and Defense Mechanisms

Beutel (1990) defines coping mechanisms as mostly *conscious*, not automatic, cognitive and experience-related or behavioural processes in persisting, aversive situations or situations expected to become aversive. Since the development of the transactional stress model by Lazarus and his colleagues (Lazarus and Launier 1981; Lazarus and Folkman 1984), coping mechanisms have become the subject of intensive psychological research (Overview: (Schwarzer 1998)). The subsequently developed category systems and instruments, such as the COPE (Kato 2015; Carver et al. 1989), made it possible to discover coping mechanisms through self-disclosure and to develop first coping concepts. However, the role of individual personality traits and (biographical) motives was often neglected in those coping concepts and scales (Steffens and Kächele 1988). Lazarus himself admitted that unconscious intentions were hardly represented in this approach (Lazarus 2000). Although research results in recent years support a high proportion of unconscious, intuitive processes, especially in complex, fast decisions (Horr et al. 2014; Gigerenzer and Kober 2009), in this respect little has changed in the approach to coping research to our knowledge.

During the last decades, the research on defense mechanisms has grown immensely. Psychoanalysts from different schools interpret defense differently, and every definition involves the risk of an over-reduction of this very complex construct. In their 600-page book on the current state of theory and research of defense mechanisms, Hentschel et al. (2004) rightly stress the complexity of the subject. They also acknowledge the often divergent attitudes to psychoanalysis in general and to defense mechanisms in particular of the psychological research field. According to Beutel, defense mechanisms can be understood as *unconscious*, primarily cognitive and experience-related processes that include a narrowing or distortion of intersubjective reality and self-perception (Beutel 1990). After the development of Sigmund Freud’s theory of defense, its further development by his daughter Anna Freud and the publication of her work ‘Ego and the Mechanisms of Defense’ (Freud 1936), the concept of defense was supplemented by intrapsychological and interpersonal perspectives of the various currents within the school of psychoanalysis. In recent decades, authors such as Vaillant, Laughlin and König have repeatedly attempted to develop and introduce a new kind of taxonomy, but the number and classification of the chosen defense mechanisms differed considerably from author to author (Vaillant 1971, 1992; Laughlin 1979; König 1997; Seiffge-Krenke et al. 2017). In addition, the concept of the individual’s psychic structure has become increasingly important in theory, in the context of operationalized psychodynamic diagnostics (OPD) (Cierpka et al. 2007; Cierpka 2014), as well as in psychotherapeutic and trauma therapy practice (Rudolf et al. 2004; Wöller 2013).

There seems to be relative agreement that defense and coping mechanisms have or are Ego-functions, with defense being used for inner-psychic protection and regulation and coping for real adaptation and problem solving (Steffens and Kächele 1988). In other words, only the corresponding (unconscious) defense enables successful (conscious) coping (Cierpka 2014).

In psychoanalytic theory and research certain character types (König 2004) and clinical diagnoses are associated with certain constellations of defense and coping mechanisms, for example the obsessive-compulsive disorder with reaction formation and isolation (Rubino et al., 2007) or the borderline personality disorder with mechanisms such as sensation seeking, autoaggressive behaviour, dissociation, splitting and many more. In recent decades, numerous well-known authors have repeatedly expressed criticism of an explicit separation of coping and defense (Cierpka 2014; Steffens and Kächele 1988; Beutel 1990). Steffens & Kächele already wrote in 1988: “We consider it sensible to give up a strict separation of coping and defense. Both processes complement each other, by no means exclude each other alternatively”. Nevertheless, in the past, there were repeated “denial efforts of kinship relations” in both concepts (Cierpka 2014; Steffens and Kächele 1988; Beutel 1990). Even now, at least in Germany, the use of either coping self-rating scales in clinical psychiatric practice and science, on the one hand, and the focus on unconscious conflicts and defense mechanisms of psychoanalytic therapists and scientists, on the other hand, indicate a continued one-sided approach. To our knowledge, there has been no practical, integrative model for the identification and representation of coping and defense mechanisms in trauma-related disorders. This deficiency is surprising since coping mechanisms (such as sports or drug consumption) and/or unconscious defense mechanisms (e.g., rationalization or splitting) play an important role in diagnosis, therapy planning and prognostic assessment by therapists of different schools. This role is especially true for patients with trauma-related disorders, in whose treatment Ego-supporting, affect-regulating interventions are often of great practical importance.

In general, active coping (e.g., fight or flight) is more likely to be used if the person assumes he/she can control the threat or escape from it. If the individual considers control or escape impossible, he/she usually reacts with passive mechanisms (Olff and Langeland 2005). It was, therefore, assumed that the use of certain coping mechanisms was primarily situation-dependent. On the other hand, there are also indications that a certain coping style or the repetitive use of certain mechanisms can represent a risk factor in the development of trauma-related disorders (Chang et al. 2003). This risk corresponds to the psychoanalytical assumption of a ‘defense and coping profile’ linked to the psychological structure and to findings from current trauma research indicating that an individual’s coping style or coping mode influences whether the affected person can successfully handle the traumatic experience or develops a mental illness such as PTSD (Chang et al. 2003; Johnsen et al. 2002).

### Goal of this Study

Apart from a phenomenological description of coping and defense mechanisms, this study aims to determine if there are specific combinations of mechanisms that lead to special adjustment types among former GAF soldiers and their approach to psychotherapeutic or psychiatric treatment.

## Method

### Research Context

This study was conducted within the framework of the “German former soldiers’ Readjustment Study”, which is a research project designed to gain insight into the daily life of former German soldiers, including their life satisfaction, economic situation, family situation and their health status. It was approved by the ethics committee of the Charité Universitatsmedizin Berlin (Approval Number EA1/250/14).

### Recruitment and Sampling

Recruiting potential participants took place via a project-owned website and the psychiatric ward of the GAF Hospital Berlin. Former soldiers of the GAF who had participated in at least one foreign assignment were included. Thus, it was possible to identify 103 potential participants, of whom five could not ultimately be contacted. Short telephone interviews were conducted with the remaining 98 participants. They were asked about their motivation for participating and to provide their socio-demographic data. The inclusion criteria were checked as well. In all, 43 of the remaining 98 participants were eventually invited to phase two of the project, the open interviews. The participants were initially selected at the Bundeswehr hospital in Berlin using opportunistic sampling (Teddlie and Yu 2007). Because theoretical saturation arose during the course of the interviews and the initial analysis, other interview partners not associated with the hospital were selected, visited and interviewed in their homes. For this purpose, participants with and without psychological symptoms and those who did or did not use psychosocial services were selected to create a contrasting sample (based on the theoretical sampling of Strauss and Corbin (Corbin and Strauss 2008)) and to achieve the highest possible degree of variation. Each interview was promptly followed by a debriefing session with the research team. Form, situation, content and countertransference experience were compared with previous interviews, and the next procedure was determined, especially with regard to sampling. After a total of 43 interviews, we reached theoretical saturation, which eventually led to the cessation of data collection.

We then subjected 16 interviews, again selected on the basis of contrasting features (origin, age, gender, relationship status, number of children, training level, military rank and organizational area, country of mission, duration of deployment, psychological symptom burden, psychotherapy experience, and claim of service-incurred disability), to a detailed thematic analysis and compared the resulting concepts with the remaining 27 interviews.

### Participants

The included sample comprised former soldiers of different ranks (from soldiers to senior officers), organizational units (Army, Navy, Air Force, Joint Support Service, and Central Medical Service) and federal states of Germany (12 out of 16). Four (9.3%) participants were female; the age span of the participants was between 26 and 69 (MW = 40.4; SD = 12.3). Some participants had just left the GAF, and others had been civilians for several years. According to the former soldiers interviewed, 19 (44%) suffered from a service-incurred disability, and three (6.9%) were in treatment time, which is a time span used for clinical and occupational rehabilitation in Germany during which soldiers, although not on active duty, cannot be discharged from military service.

### Data Collection

The data were collected in personal, open interviews. The interviews were conducted jointly by two of the authors, with one of them moderating the discussion and the other responsible for the equipment and accompanying field observations. This also included his/her own counter-transference experience and observations of emotional reactions of the interviewees and in some cases, for example, their interaction with relatives or partly present pets, etc.

During the interviews, the researchers used a narrative technique (Küsters 2014) that always started with a general introduction about the former soldiers’ experiences and their adjustment in the GAF before, during, and after their deployment, as well as after their discharge and today. The interviewers let the participants talk about whatever seemed to be significant to the participants. If inconsistencies occurred, or if emotional responses were displayed, or if challenging situations were described, the interviewer encouraged the participants to elaborate more deeply on the emerging issues, especially with regard to conscious and unconscious mechanisms to adjust. The interviews were finished when the participants had nothing left to describe. *Conscious* coping mechanisms (e.g., alcohol consumption, sport) were reported during the interview, sometimes without being asked and sometimes directly asked at a later point in time. *Unconscious* coping and defense mechanisms were identified in the very detailed descriptions of the participant’s situation and action processes (e.g., rationalization, denial) or observed during the interview (e.g., affect isolation, dissociation). The entire interview was recorded in MP3 format and then transcribed by an external transcription service pledged to confidentiality (Dresing et al. 2015).

### Data Analysis

The methodological analysis process based on the principles of grounded theory and thematic analysis involved the iterative generation of hypotheses and the development of new models and typologies. According to Chapman et al. 2015, we also approached the material iteratively through inductive development, deduction and validation to develop a theoretical model anchored in the material, whereby the data collection and analysis processes were continued until saturation occurred, which is when no new insights result from data collection and analysis. Our approach corresponded to the steps mentioned by Guest et al.: 1. getting to know the material, 2. identifying thematic categories, 3. identifying structures and combinations and 4. building a theoretical model based on the findings (Guest et al. 2012).

The first step consisted of getting to know the material by repeatedly listening to the interviews, reading the transcripts and making notes and cross-references using the MAXQDA12 program. First codes were given to note down or summarize the observed or otherwise identified coping and defense mechanisms. For example, a passage in which an interviewee described how he cried during psychological sessions was given the code “crying” (along with others). In the second step, thematic categories were identified by combining, contrasting and merging similar or interconnected coping and defense mechanism codes. For example, the code “crying” was then combined with other codes such as “expressing sadness or anger” or “showing feelings”, and the category “show emotions” was created. The third step included the review and analysis of the thematic categories found to identify underlying thematic structures, motifs, functions or specific combinations of coping and defense categories using new raw data from the ongoing data collection and analysis. To continue illustrating with the above example, the passages and codes were read again, the codes were compared, and the intensity and context in which emotions were shown or not shown were examined or other codes with emotional content were added to the category. The fourth and final step was to develop a typology based on the combinations of mechanisms found in the material, constantly cross-checking it with new material and also including current literature and existing models. Thus, for example, different manifestations of the area “emotion” arose and a model continuum with the superordinate area “emotion” was created, consisting of the three emotion categories “defensive”, “partially permissive” and “affirmative” (Tables 1 and 2).

**Table 1:**
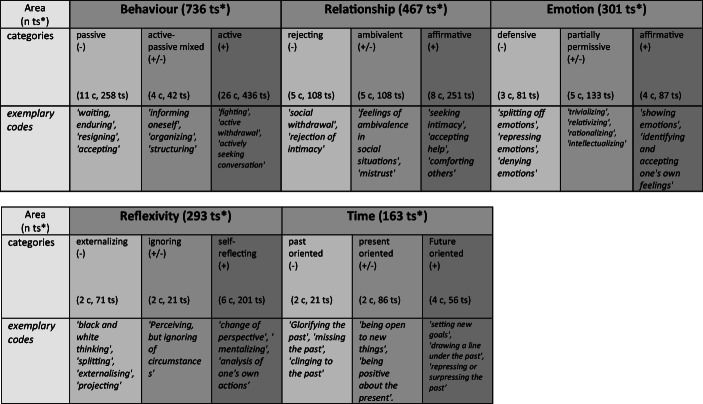
Categories, Codes and mechanisms

*c = codes, ts = text segments

**Table 2:**
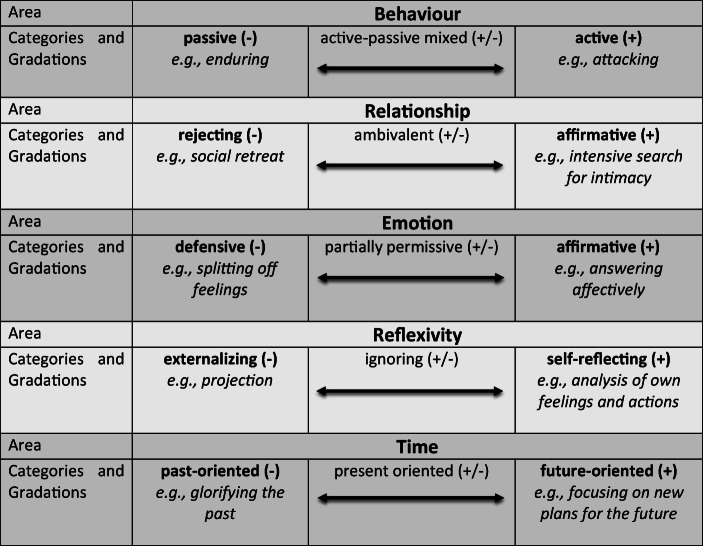
Identified coping and defense areas

For further verification and differentiation of our results, we used the “Document Portrait” function of the MAXQDA program, which is a tool for visualizing an interview by representing it as an image of its codes. The frequency and length of the coded defense and coping mechanisms within the conversation are displayed as a graphical representation, whereby the length of a segment is taken into account and included as a weighting factor for the graphical representation. The result of the preceding steps was the development of a typological classification, which we have summarized in the form of results tables for better representability (Tables 3-9).

**Tables 3:**
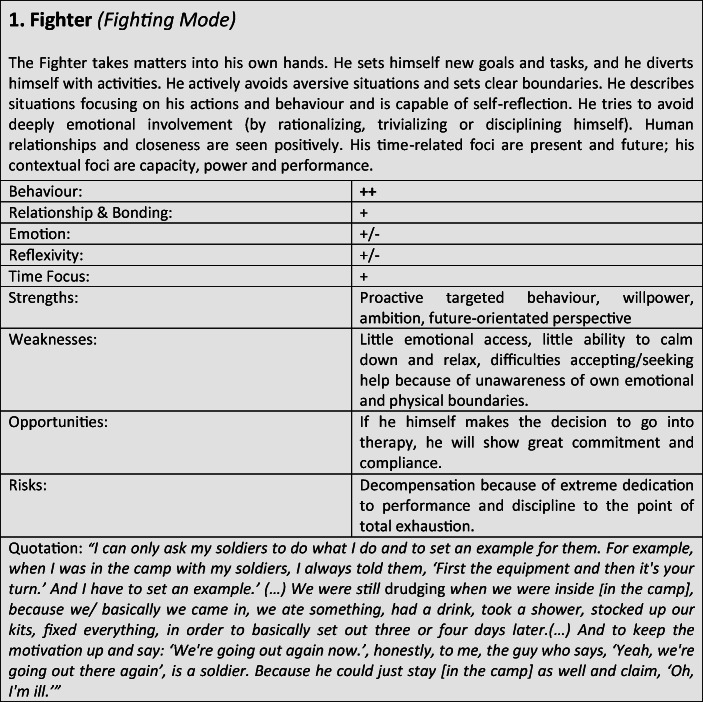
Prototypical classification

**Tables 4:**
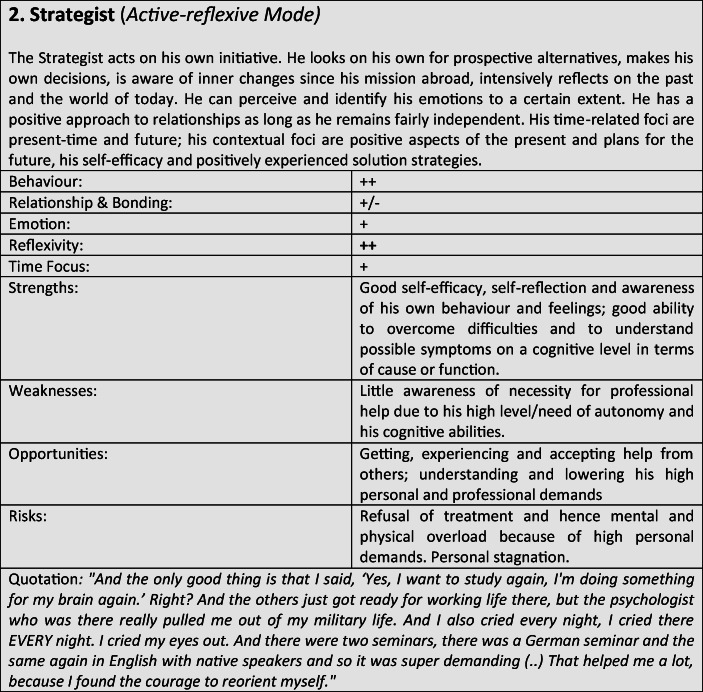
Prototypical classification

**Tables 5:**
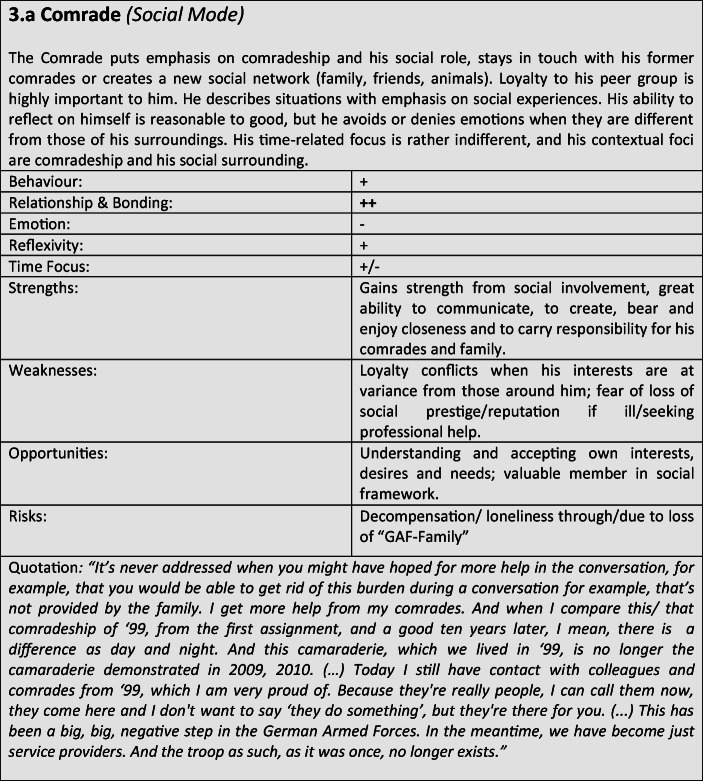
Prototypical classification

**Tables 6:**
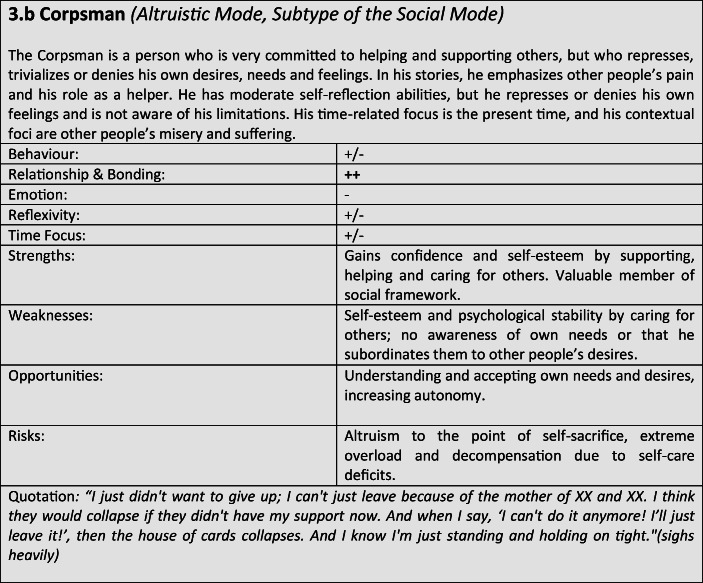
Prototypical classification

**Tables 7:**
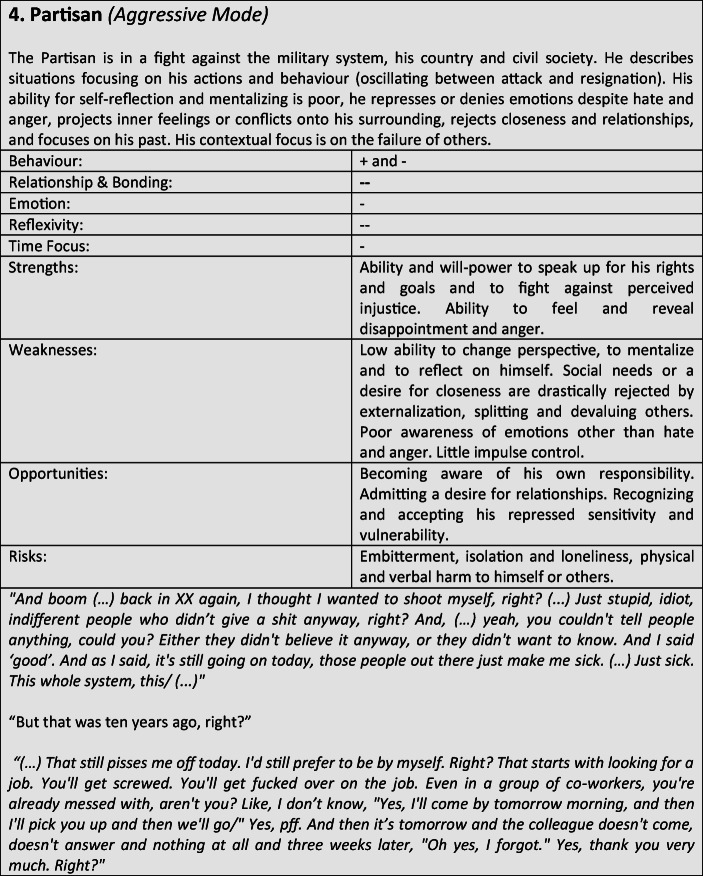
Prototypical classification

**Tables 8:**
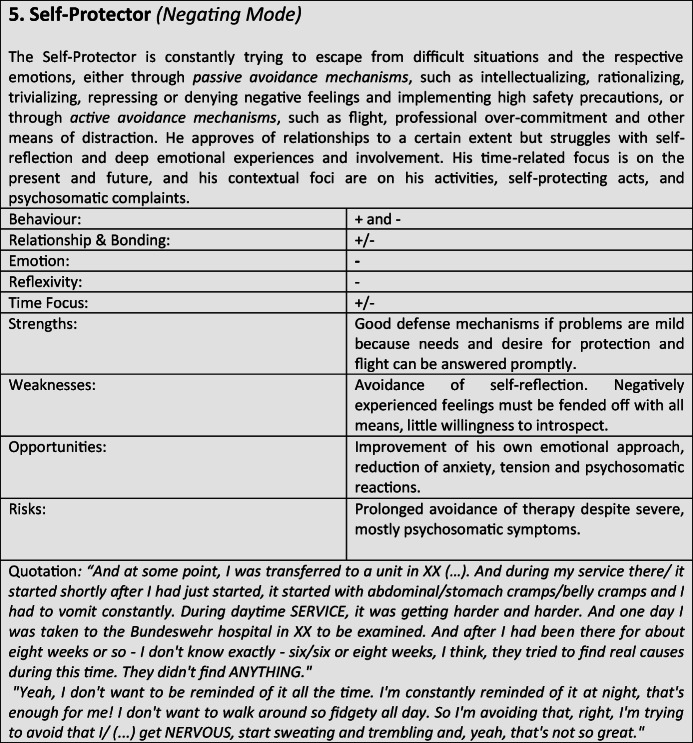
Prototypical classification

**Tables 9:**
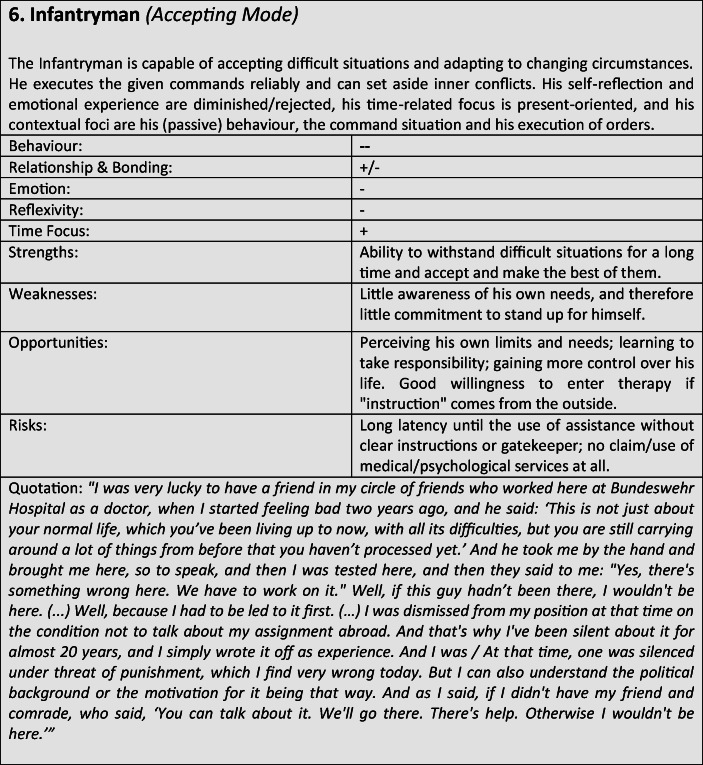
Prototypical classification

### Quality Assurance

To ensure the highest possible quality during the scientific process, qualitative research was conducted in accordance with qualitative research quality criteria (Stamer et al. 2015). In addition to the previously mentioned theory-based, iterative approach to sampling and data collection, analysis and verification, we also chose a multi-perspective, multidisciplinary research team (doctors and psychologists with and without clinical experience and with and without military background or deployment experience abroad). The validation processes in the research group, in particular, proved to be a very important element for the conscious, reflexive, flexible and self-critical handling of the data and team members’ pre-concepts. In addition to the internal communicative validation by presentation of the (partial) results and discussion of (partial) results and corrective consensus-building, all project members committed themselves to regular participation in advanced training and external research workshops and to external research supervision.

## Results

Overall, a very large number and range of described, named and observed defense and coping mechanisms were found in the group we interviewed as well as in the individual interviews. Thus, in the first step of thematic analysis (according to Guest et al. (2012) and Chapman et al. (2015)), we extracted a total of 1960 text segments containing forms of conscious and unconscious coping and defense mechanisms, which were ordered in 89 codes. In the second analysis step, these codes were categorized according to their content, motif and function and assigned to 15 superordinate coping and defense categories (See Table 1).

In the third step, the examination and analysis of the categories found to identify underlying thematic structures and combinations, the existing 15 coping and defense categories were reduced to the five overarching thematic areas of ‘behaviour’, ‘relationship’, ‘emotion’, ‘reflexivity’ and ‘time’, whereby each area could be divided into three value-neutral gradations (−, ± and +) (See Table 2).

In the fourth step, theoretical assumptions were compared with the newly found data and coping/defense modes and combinations were identified and verified using the ‘portrait function’ (see: Data analysis) and integrated into a prototypical classification (See Tables 3-9).

Although most former soldiers showed a large variety of different coping and defense mechanisms, many of the interviewees used a repetitive pattern of individual core mechanisms at different stages of adaptation. Analysing the combination, frequency and dominance of use of certain coping mechanisms in the investigated sample, seven distinguishable defense and coping types with special coping modes could be identified: *the Fighter, the Strategist, the Partisan, the Self-Protector, the Infantryman, the Comrade* and *the Corpsman*, whereby the last can be seen as a subtype of *the Comrade*. The following tables describe the various subtypes according to their prototypical traits, expressions and modes (Tables 3-9), whereby the strengths, weaknesses, risks and opportunities of the types were clarified according to the principle of SWOT-analysis. A sample quote from an interview with a participant assigned to the corresponding type, translated from German, is intended to serve as clarification. For ease of readability, the masculine form is used in the following description. However, the descriptions apply to both sexes.

## Discussion

### Reflexions and Interpersonal Aspects

When we launched our website, we anticipated a moderate response. This turned out to be a mistake. After just one week, the call to participate in the study had been shared over 28,000 times. We had underestimated the veterans’ need to communicate and their feeling of not being seen in their suffering. This impression was strenghtened in the further work process. The fact that the members of our research group were part of a well-known non-military university institution but still had military knowledge and in part military experience themselves certainly contributed to greater openness in the interviews.

When we asked the potential interviewees in preliminary telephone interviews about their motivation to talk to us, we often heard answers like: *“Finally there’s someone listening”, “For me, participating in this project is the first step in dealing with what I have experienced”*, or “*Participation is the chance that something finally changes. Most people don’t dare to speak.”* The great expectations of us and our project motivated us, but the idealization in some of those answers also made us afraid to disappoint those needs and hopes.

The interviews showed a very detailed descriptions of the behaviour and activities of the former soldiers at home and abroad. In contrast, hardly any of the interviewees spoke much about their emotional experiences. When feelings were shown openly, this often happened in the form of hardly regulated emotional reactions. The question of whether this phenomenon is a group characteristic or a symptom of trauma disorder (in the sense of emotional numbness or flooding) cannot be answered with absolute certainty. Our analysis showed that some emotions such as disappointment, anger and bitterness were frequently addressed and named, while others, such as sadness, guilt, shame and fear were hardly expressed or mentioned. One possible interpretation of this discrepancy could be that feelings such as disappointment, anger and bitterness were often directed to the external military or civil system and are also better compatible with the soldiers’ self-standard (Schuy et al. 2019). Sadness, fear or shame, on the other hand, might be feelings that are less compatible with this ideal and therefore had to be fended off unconsciously or were deliberately concealed from us. This leads to the assumption that, regardless of post-traumatic symptoms, there may be a special degree of conscious and unconscious rejection of emotions within our group.

We also have to acknowledge that the respective military rank may have an influence on the mechanisms and behaviours described. Types such as the *infanteryman*, for example, are likely to be less frequent and less long-term in leading positions, while types such as the *strategist* are more likely to feel comfortable in a leadership position on account of his need for autonomy as well as his abilities. However, these interactions certainly also exist in the civilian field.

### Classification of the Results

The typology presented here is a first classification attempt based on the data material to illustrate an integrative approach linking schools and approaches to psychotherapy to be able to make an initial assessment of the conscious and unconscious coping abilities, strengths, weaknesses and psychotherapeutic needs of former GAF soldiers within the course of diagnostic conversations without the use of further questionnaires and independent of the psychotherapeutic background of the diagnostician.

The great individual variability in coping and defense mechanisms used by former soldiers at different points in time confirms the frequently cited thesis that the situation and the perceived threat influence an individual’s choice of coping mechanisms, whereby a simultaneous use of different mechanisms usually occurs (Olff and Langeland 2005). On the other hand, we have been able to identify recurring, often unconscious, core mechanisms in individual participants, which were used at several points of time. This consistency speaks to their cross-situational use. This consistency corresponds to the psychoanalytical assumption of a ‘defense profile’ linked to the psychological structure and to findings of current trauma research indicating that the individual coping style influences whether a person successfully processes a trauma in the long term or develops a trauma-related disorder (Chang et al. 2003; Johnsen et al. 2002).

Overlaps of psychoanalytic character types and structures (e.g., König 2004) with the types introduced in this article can be seen. There are also similarities to coping concepts such as the classification of COPE (Carver et al. 1989). However, both are closely associated to specific schools of psychology and therefore are often interpreted within the respective theoretical framework. Our aim was to use an unbiased and open approach to this topic to detect underlying patterns which are not automatically associated with an existing theoretical framework and therefore enable the integration of different perspectives. Therefore, the examination of existing theoretical backgrounds took place at a rather late point in time and involved more the comparison or exploration of similarities and differences.

Revealing unconscious mechanisms requires a personal interview situation, and an external view is absolutely necessary. The research results of the last few years underscore the high proportion of unconscious, intuitively functioning processes, especially in the case of complex, fast-track decisions (Horr et al. 2014). It is virtually impossible to record such processes by filling in self-rating scales, such as coping scales such as the COPE (Carver et al. 1989). In fact, only on the basis of our interview approach were we able to identify additional mechanisms, to differentiate more precisely between emotional and reflexive reactions and to recognize the motives, gradations and characteristics of relationships, emotional approval, or reflexivity. Only in the context of conversations could motives be identified and coping mechanisms be detected and strictly assigned to the corresponding specific categories and modes.

Despite scientifically well-investigated and effective therapy methods, it is well-known that only a small percentage of (former) soldiers with mental illnesses use psychosocial services in the USA, Great Britain and Germany (Hoge et al. 2004; Stecker et al. 2007; Murphy and Busuttil 2015; Iversen et al. 2010; Siegel et al., 2017), and many wait for years to decades before entering treatment (Sayer et al. 2009; Sayer et al. 2004; Wang et al. 2005; Sayer et al. 2007). Even if they see a doctor, many evidence-based treatment options that work in other clinical contexts often seem to fail in this specific context. One reason may be that members of the military system have different basic assumptions about mental illness and psychotherapeutic treatment from civilian patients. The conflict of identity that arises in the soldier from the implicit weakness of the mental illness and the fear of stigmatization may be even greater than in members of civilian society (Schuy et al. 2018).

Rüsch et al. also emphasized the relevance of fear and experience of stigmatization and discrimination among soldiers in their qualitative work on the self-revelation of psychiatric disease (Rüsch et al. 2017). This finding raises the question of whether it is time to take a different approach. Taken these findings into consideration, our typology allows for a more tailored approach to psychotherapy and help. This approach could be a way to increase willingness to seek help and establish a relationship with the patient. Referring to the typology we introduced, it might be sensible to ask whether, for example, social and altruistic types such as *the Comrade* or *the Corpsman* are more likely to engage in therapy, regardless of psychotherapeutic school, if they are offered a group therapy setting at the outset or by offering a sports therapy programme to active types such as *the Fighter*, who quickly feel insecure in emotional contexts and use mechanisms, such as rationalization, trivialization, fighting, and powering out. Such a programme might be more inviting and would approach those former soldiers on their own terms before they are transferred to a more traditional therapeutic approach that focuses on communication and emotional insights, an approach with which those types might otherwise not be able to engage before their suffering has increased immensely. Individuals who may have internalized a very rigid male soldier ideal could, in this way, be slowly introduced to emotional topics. A subsequent psychodynamic or cognitive behavioural (psychotrauma) therapy, in accordance with proven guidelines and recommendations, would then be the second step. The same may be true of *the Partisan*, who would most likely benefit most from a mentalization-based therapy since it seems to be (psychoanalytically expressed) more a structural problem that prevents him from leading a balanced and successful life than a question of conflict.

We are fully aware of the fact that our attempt at type formation is only a first step that must be confirmed by further research. However, we believe it is necessary to develop new approaches to mental illness and the use of therapy for former GAF soldiers in order to counteract the suffering of individuals and their families. Due to the high number of unreported cases of mentally ill soldiers in need of treatment (Wittchen et al. 2012) and the growing number of traumatized individuals in recent times, we consider a new approach to be overdue.

We know that seeking help is not always a conscious, cognitive decision. In the decision process, many subconscious notions and stereotypical assumptions play a role and influence the outcome of this decision-making. Our typological classification creates initial insights into underlying motivations of conscious and unconscious coping and defense mechanisms and might be helpful in the adaptation of help on offer and therapy for the specific needs and abilities of various former soldiers. Additional research on conscious and unconscious coping mechanisms in trauma-related disorders is necessary independent of our results. From the point of view of care research, for example, the identification of individual core mechanisms, ideally at different examination times, could shed light on the influence of traumatic experiences on personal coping and defense styles and be used in the long term to create specific therapy regimes or for a more nuanced examination of the question of personal vulnerability.

The question also arises as to how far the types identified can also be found in other socio-cultural contexts. Are there such types in the American military, too? Would it not make sense to also consider an appropriate typology for the treatment of civilian patients with trauma-related disorders? After all, everyone has their own coping (and defense) mechanisms. Would it be an opportunity for all groups who find therapeutic access difficult to think about including coping and defense much more consciously in their therapy planning? This approach would mean that coping styles could be used to enable therapy by changing and reducing the individual’s defenses.

### Critical Discussion of the Typological Construct

Although critical opinions of typological constructs must be considered, it has to be stated that many of these concepts are well-known and are currently widely used, such as the Myers-Briggs-Test or the concept of Typus Melancholicus (Kronmüller et al. 2005). Many of the theoretical models and classification experiments by famous scientists such as Karl Jaspers and Kurt Schneider are based on typological constructs and have made their way into some parts of today’s classification systems such as the DSM-IV (Jäger et al. 2016; Schäfer 2001).

The development of typologies has always been of great importance for better understanding and categorization of human behaviour, especially in the psychiatric context. According to Schäfer 2001, today the term ‘type’ is not a quantifiable scale that is used for the determination of individual characteristics. It instead represents a multi-dimensional prototype of patterns with which the individual can be compared. This comparison can be made via the endpoints of the continuum (extreme variants) or via gradual gradations on the continuum (accumulated types). In Anglo-American countries, the concept of type is given far less importance in personality research than in Central Europe. If the term ‘type’ is used at all, it is used in special cases. Especially in personality research, in the Anglo-American world, the term ‘personality trait’ is preferred to ‘personality type’. As these ‘traits’ also occur with different degrees of expression and have no fixed boundaries, they are nonetheless very similar to the ‘types’ in many respects (Schäfer 2001).

Compared to categorical or dimensional approaches, typologies enable scientists and practitioners to use abstraction and reduction to make a complex process understandable and descriptive. In the past, critics of typological approaches rightly criticized the frequent lack of differentiation and validity of typological constructs. In relation to the past, this criticism is not completely unjustified. However, there are also counterexamples to the charge of lack of validity, such as the theory of personality functioning by Block and Block, who divided individuals into’ resilient’,’ over-controlled’ and’ under-controlled’ (Block and Block, 1980). The validity of the model was statistically confirmed in several studies (Bohane et al. 2017). Regardless, typological analysis is ideally carried out in three steps: 1) finding the types, 2) describing the types and 3) diagnosing the types using qualitative *and* quantitative methods (Zerssen 1973). We, therefore, see our attempt to identify defense and coping mechanism types in the group of former GAF soldiers as a first step in the generation of hypotheses (finding and describing the types), which will hopefully be followed by more qualitative and quantitative validation steps in the future.

### Strengths and Limitations

The study is a pilot study for the identification, illustration and typological classification of conscious and unconscious coping mechanisms of former soldiers with deployment experience. With 43 interviews, the study has a broad database for a qualitative design. Due to the high media resonance and the additional recruitment in the hospital and within the military, we likely reached a very large number of former soldiers and included a large variability of contrasting cases through the differentiated sampling. Influences by the investigator and resulting psychodynamic processes were minimized by the sampling, the divergent team composition (medical doctors and psychological staff of both sexes with and without military and combat experience and with and without psychotherapeutic training in different psychotherapeutic schools), and analysed and included in the evaluation by the conscious handling of counter-transference experiences and regular team intervention and supervision.

The open nature of the interview design with its low limitations, the lack of requirements and the possibility of asking questions led to very detailed, individual, subject-related descriptions of the internal and external processes. For most of the participants the interview situation, especially the conversation about stressful events in the war zone, was a challenge in itself. Therefore, some defense and coping mechanisms were already apparent during the interview which we were able to experience “live”. Through the shown or not shown affect, the choice of words, the tone of voice and they kind of description, mechanisms were already recognizable in the interview situation. Our approach therefore involved, on the one hand, the analysis of the mechanisms remembered and narrated by the participants themselves, and, on the other hand, the consideration of the unconscious mechanisms and behaviours recognisable from the narrative. By the additional analysis of those mechanisms directly occurring in the course of the interview, we believe we counteracted a conscious or unconscious selection or distortion process, as occurs in any form of (retrospective) survey. In other words, these phenomena helped us to better understand the (unconscious) fears, values and motives of the former soldiers interviewed.

Although we have paid great attention to differentiated sampling with the aim of achieving the greatest possible heterogeneity and have always oriented our data collection and analysis to the quality criteria of Stamer et al. 2015, there is still no need for generalizability in the sense of statistical representativeness, as is generally not the case with any form of qualitative procedure. Our typological classification constitutes the preliminary results and theoretical model from a pilot study. It must and should be validated and critically reviewed, and evaluated for its practical usability, in follow-up studies.

## Conclusion

This work strives for the first time, to our knowledge, to develop a typological classification of the use of conscious and unconscious defense and coping mechanisms on the basis of methodically and structurally collected and analysed data from a qualitative pilot survey of former soldiers in Germany. Seven coping and defense types were identified: *the Fighter, the Comrade, the Corpsman, the Strategist, the Partisan, the Self-Protector* and *the Infantryman*. The types identified differed in the accumulation, combination, and use of their conscious and unconscious defense and coping mechanisms in categories in the superordinate areas of *behaviour, relationships, emotions, reflexivity* and *time focus*. The typological classification could offer psychotherapeutic interventions tailored to individuals and their defense and coping mechanisms, which could lead to improved therapy use and compliance. Nevertheless, further research is needed in the field of trauma management and validation and verification of our results in follow-up studies.
